# Ablation of Bone Tissue by Femtosecond Laser: A Path to High-Resolution Bone Surgery

**DOI:** 10.3390/ma14092429

**Published:** 2021-05-07

**Authors:** Laura Gemini, Samy Al-Bourgol, Guillaume Machinet, Aboubakr Bakkali, Marc Faucon, Rainer Kling

**Affiliations:** ALPhANOV, Institut d’optique d’Aquitaine, 33400 Talence, France; samy.al-bourgol@alphanov.com (S.A.-B.); guillaume.machinet@alphanov.com (G.M.); aboubakr.bakkali@alphanov.com (A.B.); marc.faucon@alphanov.com (M.F.); rainer.kling@alphanov.com (R.K.)

**Keywords:** femtosecond laser, bone tissue ablation, process upscaling

## Abstract

Femtosecond lasers allow for high-precision, high-quality ablation of biological tissues thanks to their capability of minimizing the thermal loads into the irradiated material. Nevertheless, reported ablation rates remain still too limited to enable their exploitation on a clinical level. This study demonstrates the possibility to upscale the process of fs laser ablation of bone tissue by employing industrially available fs laser sources. A comprehensive parametric study is presented in order to optimize the bone tissue ablation rate while maintaining the tissue health by avoiding excessive thermal loads. Three different absorption regimes are investigated by employing fs laser sources at 1030 nm, 515 nm and 343 nm. The main differences in the three different wavelength regimes are discussed by comparing the evolution of the ablation rate and the calcination degree of the laser ablated tissue. The maximum of the ablation rate is obtained in the visible regime of absorption where a maximum value of 0.66 mm^3^/s is obtained on a non-calcined tissue for the lowest laser repetition rate and the lowest spatial overlap between successive laser pulses. In this regime, the hemoglobin present in the fresh bone tissue is the main chromophore involved in the absorption process. To the best of our knowledge, this is the highest ablation rate obtained on porcine femur upon fs laser ablation.

## 1. Introduction

In the last decade, ultra-short-pulse lasers gained a unique place in several industrial sectors thanks to the readiness of the laser technology, which can nowadays guarantee competitive processing time for most laser processes together with the unmatched processing quality and high resolution achievable with these types of laser sources [1]. Moreover, the possibility to deliver the laser beam through adapted optical fibers opens the way to processing of complex 3D parts or shapes by employing robotic arms guided remotely by specific software or directly by hand-guided pointers [2]. In this context, the implementation of such systems has attracted growing interest for several biomedical applications where high precision and highly localized intervention are needed [3,4,5]. For instance, in the frame of bone tissue removal in surgical operations such as craniomaxillofacial surgeries, it is favorable to reach high-precision ablation while keeping the surrounding tissue the healthiest in order to favor the regeneration and the overall recovery of the tissue following the surgery. Femtosecond (fs) laser ablation is well-known to be characterized by negligible thermal effects on the material upon laser–matter interaction thanks to the very short laser pulse duration, which ends before the electrons may have time to thermalize and transfer the absorbed laser pulse energy to the lattice [6,7,8,9]. This specific property is of extreme importance when considering the interaction of light with biological tissues because the death of such tissues is a direct consequence of the tissue temperature increase and strongly prevents the tissue regeneration and the overall after-surgery recovery [10].

The main issue linked to the full exploitation of such systems in the clinical environment remains the low ablation rate which can be achieved with respect to classical techniques such as mechanical approaches or even other types of laser sources [7,9,11]. Laser systems employed nowadays in the clinical environment for applications including enamel ablation in dentistry, skin ablation in dermatology and bone surgery are mainly quasi-continuous wave (QCW) or continuous wave (CW) Er:YAG laser systems running at a wavelength of 2.94 µm [12]. This wavelength lies, in fact, in the vicinity of a strong absorption peak of water and hydroxyapatite, leading to high ablation efficiencies of both hard and soft tissues [13]. Hydroxyapatite is indeed a naturally occurring mineral form of calcium apatite, and in different modified forms, it represents up to 70% of the weight of human bone [14]. While bone tissue ablation rates comparable to those of mechanical approaches can be achieved with these types of laser sources, ablation remains often accompanied by important carbonization, calcination and tissue damage due to the QCW/CW characteristic of these laser sources [15]. The same detrimental thermal effects were observed when a 200 ns–532 nm laser source was employed on porcine mandible samples, while no thermal damage was visible upon irradiation with 200 fs–775 nm laser light [16]. While the unique capability of ultra-short-pulse lasers in generating non-thermal interactions in bone tissue has been largely shown in the literature, the same studies demonstrate difficulties in reaching competitive ablation rates when ultra-short-pulse lasers are employed for bone tissue ablation [17]. Moreover, the different experimental conditions, together with the strong dependance of the results from the specific characteristics of the analyzed bone tissue, such as the type of bone or animal, the age of the animal as well as the process environmental conditions, make it difficult to carry out a solid comparison for common processing conditions [18,19]. Table 1 summarizes the state of the art found in the literature on the ablation of bone tissue by ultra-fast laser sources. The highest reported ablation rate is around 0.99 mm^3^/s for sheep shank bone obtained with a 230 fs–1030 nm laser source in specific gas-cooling conditions.

In this work, industrially available fs laser sources were employed with the goal of optimizing the ablation rate by demonstrating the possibility to upscale the process of bone tissue ablation. Three different wavelengths (1030 nm, 515 nm, 343 nm) were investigated within an upscaling approach by increasing the laser average power and laser repetition rate. Thermal effects on the bone tissue linked to an increase in laser average power were analyzed with respect to the thermal accumulation by variation in laser parameters as the spatial overlap between successive laser pulses. The condition of the bone tissue after laser processing was evaluated by scanning electron microscopy (SEM) and energy-dispersive X-ray (EDX) analyses to qualitatively observe morphological changes as well as to quantify the degree of laser-induced calcination. Results show the predominant role of thermal accumulation in the calcination process of bone tissue and demonstrate the possibility to employ industrially available fs laser sources within an upscaling approach to achieve competitive ablation rates with respect to the existing literature values.

## 2. Materials and Methods

### 2.1. Preparation of Biological Samples of Porcine Femur

Several studies can be found in the literature on the assessment of specific interspecies differences in bone tissue and a common agreement is reached in stating that a unique animal model able to reproduce the human situation does not exist. Nevertheless, similarities can be found in some cases: for specific joints and tissues, the porcine model exhibits homology with humans in terms of anatomy and/or biomechanics [18,24,25,26]. In this work, several porcine femurs were collected from the same butcher and labeled with the age and sex of the animal. The femurs were conserved at a temperature of −6 °C. Before treatment, they were left defrosting for around 1 h at room temperature in a laminar air-flow protection system (DE HERAsafe KS12, Thermo Scientific, Waltham, MA, USA) and cleaned with a scalpel in order to remove the marrow and all soft tissue. The remains of fat were removed by a pre-cleaning step in a solution of deionized (DI) water (Socimed, Stains, France) and 70% ethanol (Socimed, Stains, France). The samples were then left to dry at room temperature for around 1 h. Finally, the femurs were mechanically cut by a diamond blade (Dremel, Racine, WI, USA) in order to obtain smaller-sized samples (approx. 1 × 1 cm^2^) for easier handling during the processing and characterization steps. All samples employed for the laser processing were obtained from the diaphysis section of the femurs.

### 2.2. Experimental Setup and Laser Processing Station

**Figure 1:**
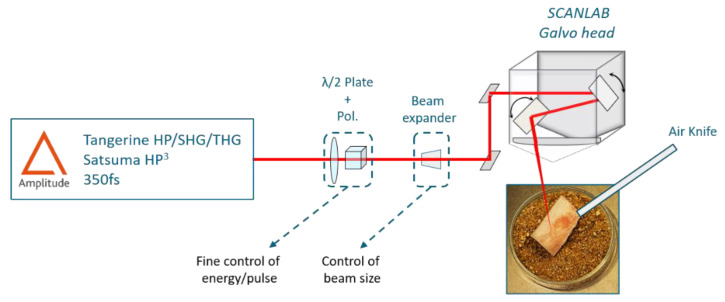
Schematic of the experimental setup used for the ablation tests.

Two different laser sources from Amplitude were employed for the tests. A Satsuma HP3 (maximum average power 40 W, maximum repetition rate 1 MHz) running at a central wavelength of 1030 nm was employed for all tests in the IR regime. A Tangerine (maximum average power 20 W, maximum repetition rate 1 MHz) running at a central wavelength of 1030 nm was coupled to a specific module able to convert the fundamental wavelength to its second (515 nm) and third (343 nm) harmonic, thus allowing operation in visible and UV regimes. The pulse duration for both sources was comparable and in the order of 350 fs. Figure 1 presents a schematic representation of the experimental setup employed for the tests. The laser beam was firstly sent through a couple of a half-wave plate and a polarizing cube for the fine control of the energy per pulse. Before entering the scanning system, the laser beam size was adjusted by a specific telescopic system in order to achieve a focused beam diameter at the output of the final focusing f-theta lens of around (2ω_0_) = 25 μm for all tests, where ω_0_ is its radius. The focused beam diameter was measured by CCD camera analyses (WinCamD, DataRay Inc., Redding, CA, USA). The laser beam was deflected and positioned by a galvanometric scanning head and finally focused on the surface of the sample by a 100 mm f-theta lens. An air-knife was employed to efficiently remove the ablation dust and at the same time cool the bone tissue during the laser processing. Areas with a dimension of 3 × 4 mm^2^ were processed on the bone samples with a scanning geometry composed of overlapping lines with inter-line distance h, each line being composed of overlapping laser pulses with inter-pulse distance d. The distance d between two successive pulses is given by the ratio between the velocity v at which the scan head deflects the beam on the sample surface and the laser repetition rate RR: d = v/RR. In all tests, the scanning speed v together with the laser repetition rate RR were varied in order to keep the distance between neighbor pulses d = h for the sake of homogeneity of the set of laser-induced effects. Finally, in order to obtain ablation cavities characterized by measurable ablation depths within the limit of the lens focal depth, a number of 10 successive passes was selected for all tests. Table 2 summarizes the intervals of process parameters employed for the laser processing in the three different absorption regimes: IR-1030 nm, visible-515 nm and UV-343 nm, respectively. For each regime, three different laser repetition rates RR were investigated up to the maximum value exploitable from the laser system. For each repetition rate RR, the average power was increased within the limit of visible calcination of the bone tissue. For each considered value of average power, the scanning speed v was varied in order to have d = h. The percentage spatial overlap OL between neighbor pulses can be then defined as OL = (2ω_0_−d)/(2ω_0_).

### 2.3. Protocols for Sample Characterization after Laser Processing

For characterization purposes, laser-processed samples were firstly dehydrated by multi-step baths in ethanol solutions according to the following procedure: 70% ethanol at 4 °C for 24 h, 90% ethanol at 4 °C for 1 h and finally 100% ethanol at 4 °C for 1 h.

Three different characterization techniques were employed on all processed samples:

- Optical microscopy (MF-B1010C, Mitutoyo, Kanagawa, Japan) analyses were carried out to evaluate the ablation depth. Five measurements were carried out on each ablation cavity in order to have statistically valid values and compensate for possible ablation inhomogeneities on the bottom of the cavities. The standard deviation was calculated to be between ±0.02 mm and ±0.09 mm for all measurements in all regimes of ablation. For all ablated samples, the ablation rate was calculated as the ratio between the ablated volume (mm^3^) and the processing time (s).
- SEM (Vega3, Tescan, Brno, Czech Republic) observations were carried to analyze the quality of ablation and the overall aspect of the bottom of the cavities in order to define a possible correlation between the observed morphology and the calcination state of the sample.
- EDX (Bruker US Quantax, Billerica, MA, USA) measurements were carried out on all processed samples in order to quantify the laser-induced variation in atomic% of the main bone tissue components, such as C, O, Ca, Mg and P. To account for the semi-qualitative nature of the EDX analyses, all measured values were normalized with respect to the reference value of the same element measured on a non-laser-processed area of the same sample. For each ablated cavity, three measurements were carried out for statistical purposes.

The state of the laser-processed bone tissue after laser interaction was defined for each cavity by two different evaluation methods as the combination of two different criteria: a visual evaluation based on the visual aspect of the ablated cavities led to a damaged/healthy assessment while the EDX analyses led to a quantifiable threshold over which the bone tissue is considered as thermally damaged. Given the sudden and quick rise in temperature experienced by the bone tissue following the laser interaction, initially, a carbonization of the tissue characterized by an increase in C content and typical blackening of the material takes place [27,28]. Nevertheless, this phenomenon occurs on such a short time scale that it cannot be observed on any laser-ablated area at the end of the laser process. Indeed, the thermally damaged samples were all left calcified after the laser processing—that is, with lower or negligible content of the volatile elements, such as C. The calcified areas were characterized by the typical grey/white color of the mineral phase of the tissue. The ratio C_BEFORE_/C_AFTER_ between the atomic% of carbon C before and after the laser process was calculated and a threshold of C_BEFORE_/C_AFTER_ = 5 was defined to discriminate a healthy tissue from a calcified one for all samples.

## 3. Results

Results on the evolution of ablation rate and calcination state of processed samples are presented separately for each wavelength employed for the laser processing. A quantitative comparison between results obtained in the different regimes is presented to support the discussion of the results in Section 4.

### 3.1. Porcine Femur Laser Ablation in IR Regime (1030 nm)

**Figure 2:**
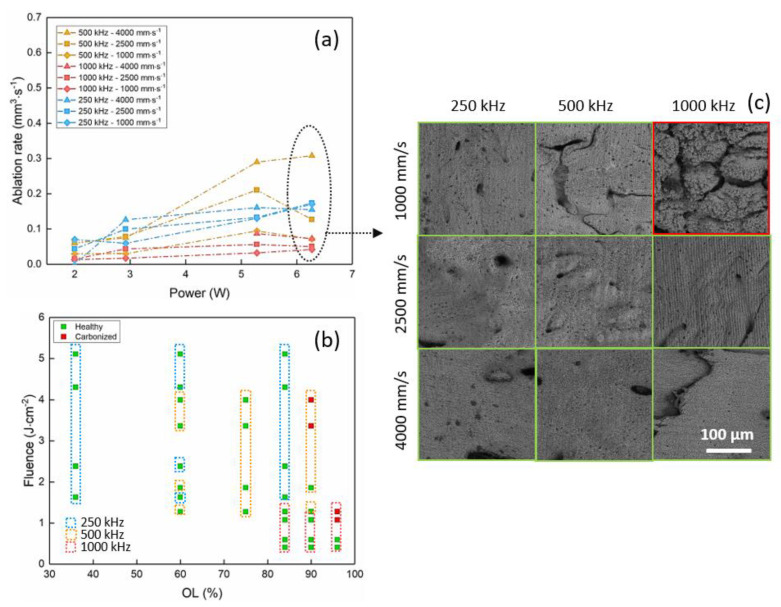
Results in the IR regime (1030 nm). Evolution of (**a**) the ablation rate with the average power and of (**b**) the fluence with the spatial overlap. (**c**) SEM images show the ablated cavities at P = 6.27 W for the process parameters indicated in the picture. Red and green squares identify calcined and healthy bone tissue, respectively.

Figure 2 shows the evolution of the ablation rate with the average power (Figure 2a) and of the fluence with the spatial overlap OL (Figure 2b). SEM images of ablated cavities at P = 6.27 W for the different process parameters are presented in Figure 2c.

It is possible to observe that for all repetition rates, the ablation rate increases with the average power up to a certain value, after which it tends to saturate. This behavior highlights the onset of particle shielding effects after a certain level of energy per pulse, which leads to a strong decrease in the ablation efficiency. An increase in the ablation rate with the repetition rate is observed up to 500 kHz, at which the maximum measured ablation rate is around 0.3 mm^3^/s. A systematic increase in the ablation rate is also observed for higher values of scanning speed.

At higher repetition rates, incubation-related thermal effects become too important, leading to a fundamental modification of the bone tissue structure and a strong decrease in the ablation rate. This observation is clearly visible in the SEM image of Figure 2c, where green and red frames are used to identify healthy and calcined ablated cavities, respectively. The calcined cavity presents large cracking and high rugosity with respect to ablated cavities, which were left undamaged by the laser processing. Results presented in Figure 2b show the effect of laser-induced thermal accumulation on the bone tissue. A strong calcination driven by an increase in the tissue temperature is connected to high spatial overlap values rather than high values of energy per pulse.

### 3.2. Porcine Femur Laser Ablation in Visible Regime (515 nm)

**Figure 3:**
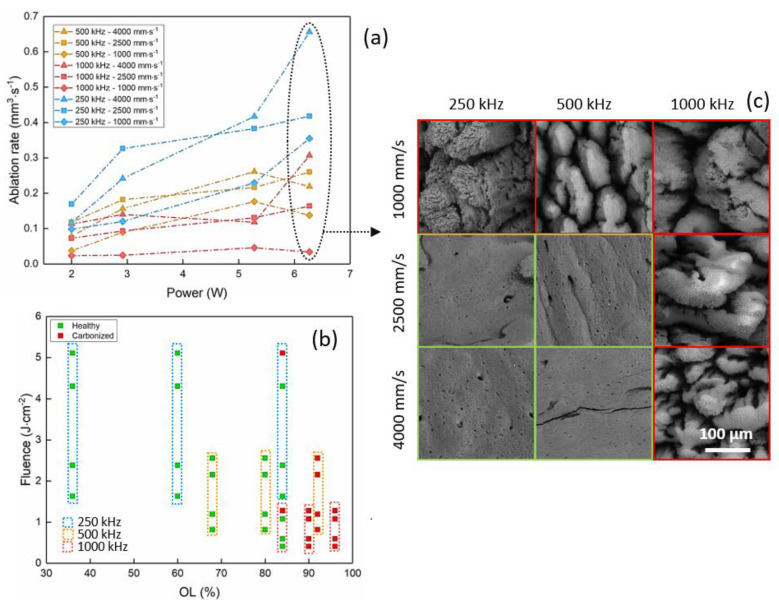
Results in the visible regime (515 nm). Evolution of (**a**) the ablation rate with the average power and of (**b**) the fluence with the spatial overlap. (**c**) SEM images show the ablated cavities at P = 6.27 W for the process parameters indicated in the picture. Red and green squares identify calcined and healthy bone tissue, respectively.

A few differences and analogies were observed for the results in the visible regime. Figure 3 presents the evolution of the ablation rate with the average power (Figure 3a) and of the fluence with the spatial overlap OL (Figure 3b). Figure 3c shows SEM images of the ablated cavities at P = 6.27 W.

As observed in the IR regime, regardless of the repetition rate, an increase in the ablation rate is measured for an increase in average power until a saturation is reached. A maximum ablation rate of around 0.66 mm^3^/s is obtained at 250 kHz for the highest scanning speed value. In the visible regime, the ablation efficiency drops at lower repetition rates with respect to the evolution observed in the IR regime. This behavior is linked to the observation that, in this regime, the bone tissue carbonizes more easily because of the efficient absorption of specific bone tissue components, such as hemoglobin and melanin, at this wavelength. At higher repetition rates, the ablation rate drops significatively and it is the lowest for RR = 1000 kHz. Laser-induced calcination and thermal effects are clearly observable also in the SEM images presented in Figure 3c: large cracks and the appearance of spherical microparticles related to thermal mechanisms are visible.

### 3.3. Porcine Femur Laser Ablation in UV Regime (343 nm)

The UV regime was investigated by converting the fundamental laser wavelength of 1030 nm to its third harmonic at 343 nm. Due to the low conversion efficiency of this process, modifications of bone tissue in this regime were studied for a limited interval of process parameters up to an average power of 3.9 W.

**Figure 4:**
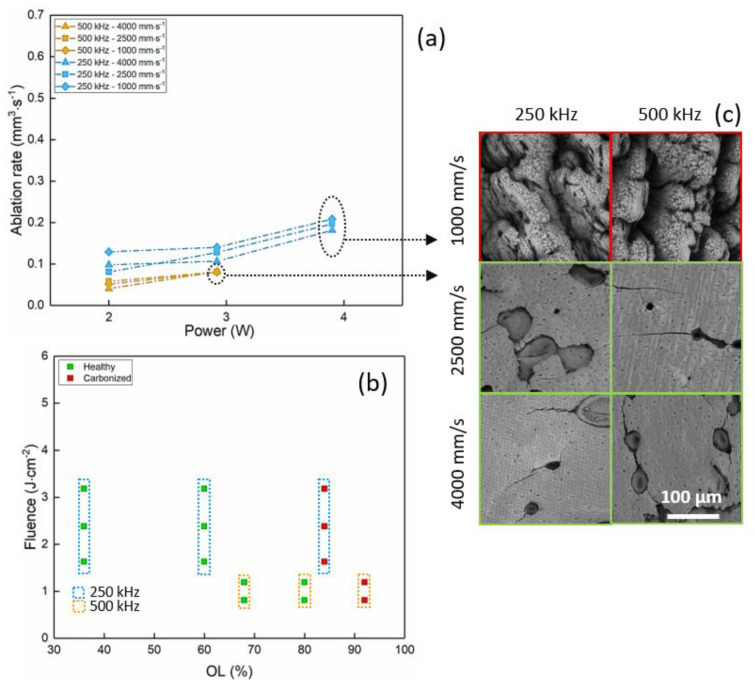
Results in the UV regime (343 nm). Evolution of (**a**) the ablation rate with the average power and of (**b**) the fluence with the spatial overlap. (**c**) SEM images show the ablated cavities at P = 2.92 W and 3.9 W for the process parameters indicated in the picture. Red and green squares identify calcined and healthy bone tissue, respectively.

Results on the evolution of the ablation rate and the calcination state after laser processing with a wavelength of 343 nm are comparable and complementary to the ones obtained in IR and visible regimes. As presented in Figure 4a, the ablation rate increases with the average power and reaches its maximum of 0.21 mm^3^/s at 250 kHz and 3.9 W. Comparing the results for the three wavelengths at the same average power of P = 2.92 W, the maximum ablation rate obtained in the UV regime is around 0.14 mm^3^/s, while it reaches up to 0.33 mm^3^/s in the visible regime and it is of the same order in the IR regime (0.13 mm^3^/s). Contrary to the results in IR and visible regimes, no particular behavior is observed with respect to the variation of the scanning speed. Calcination of bone tissue in the UV regime is observed at low pulse energy and high spatial overlap between successive laser pulses already at 250 kHz (Figure 4b,c).

## 4. Discussion

**Figure 5:**
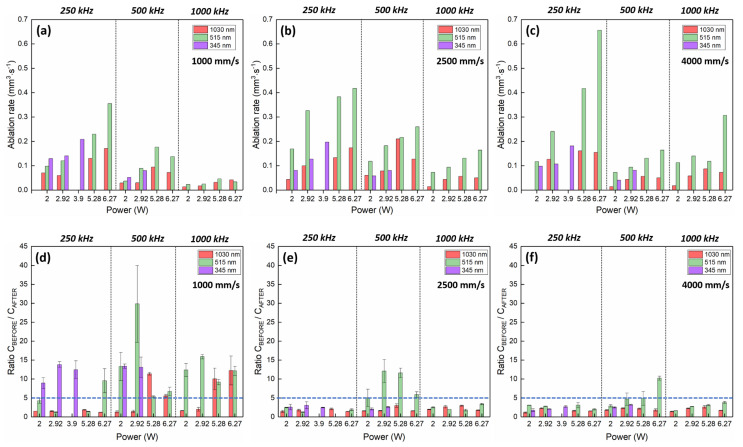
Evolution of the ablation rate (**a**–**c**) and of the ratio C_BEFORE_/C_AFTER_ (**d**–**f**) with the average power P in the three wavelength regimes (IR—red bars, visible—green bars, UV—purple bars) for all repetition rates RR (250 kHz, 500 kHz, 1000 kHz) and all scanning speeds v (1000 mm/s, 2500 mm/s, 4000 mm/s). The blue dotted line represents the threshold over which the ablated bone is considered as completely calcined.

In order to facilitate the comprehension of results, the evolution of the ablation rate and of the ratio C_BEFORE_/C_AFTER_ with the average power is visualized as histogram bars in Figure 5 for all wavelength regimes, all repetition rates and all scanning speeds investigated. Table 3 summarizes the set of laser process parameters obtained in the three wavelength regimes where the ablation rate is maximized without bone tissue damage. It is easy to highlight that the best set of laser processing parameters is obtained in the visible regime, at the lowest repetition rate and the highest scanning speed, where it is possible to achieve higher ablation rates while keeping the bone tissue healthy.

A few similarities are found between the three wavelength regimes. For a given repetition rate, the ablation rate always increases up to a certain wavelength-dependent value with the average power, reaching saturation at a given energy per pulse. This behavior is typical for ultra-fast laser ablation processes and is strongly connected to the characteristic energy penetration depth of each material [29]. For the same reason, an optimal repetition rate exists over which the ablation rate drops below its optimal value. For a given average power and a given wavelength, a decrease in repetition rate is accompanied by an increase in energy per pulse: the ablation rate increases consequently up to a point where most of the laser energy is dissipated into the material outside the energy penetration volume, where the laser energy cannot be efficiently employed to activate the ablation phenomenon [30,31,32]. In this work, the optimal repetition rate is found to be 500 kHz for the IR regime and only 250 kHz in the visible and UV regimes (Figure 5a–c). This observation can be linked to the different responses of the bone tissue chromophores in the three wavelength regimes, which leads to different ablation threshold fluences, different absorption depths and overall different ablation phenomena. In the IR regime, absorption is driven by water and hydroxyapatite contents, while in the visible regime, the hemoglobin present in the fresh bone tissue is the main chromophore involved in the absorption process. Finally, for the UV wavelength, the principal absorbers are mainly collagen and hemoglobin as well [33]. In this regime, at high cumulative energies delivered to the bone tissue, a peroxidation reaction occurs in erythrocytes during which reactive oxygen species (ROS), H_2_O_2_ and hydroxyl radicals are produced. These oxidization products will attack the hemoglobin bonds and, as a result, a denaturation reaction occurs in the hemoglobin component of the tissue [34,35]. However, the composition of bone tissue and the distribution of its absorbing chromophores are strongly dependent on the specific bone tissue characteristics, such as age, sex and type of bone, which makes it difficult to identify a comprehensive optical response for the three different wavelength regimes with respect to their energy penetration depths applicable as a general rule [18,36,37].

The role of thermal accumulation and particle shielding is noticeable by observing the drop of the ablation rate with (i) the decrease in the scanning speed for a given repetition rate—that is, an increase in the spatial overlap between successive pulses—and (ii) the increase in the repetition rate for a given scanning speed, both in the IR and visible regime. For a fixed repetition rate, the slower the scanning speed, the spatially closer the imping laser pulses are, and the higher the thermal load deposited per unit of surface into the material volume. If the repetition rate is then increased from 250 kHz to 1000 kHz, two other phenomena potentially occur: the successive incoming laser pulse might interact with the expanding plasma plume produced by the previous pulse, which could prevent its efficient absorption; moreover, the laser pulses would arrive on the surface before the irradiated material would have the time to completely thermally relax, thus leading to thermal accumulation-related effects.

Laser-induced thermal effects are detrimental in the first place for the optimization of the ablation rate and also the key factor driving the calcination of the tissue. Consequently, as the results show, a combination of a limited repetition rate together with high scanning speed leads to the optimization of the ablation rate in the three wavelength regimes. Specifically, in the IR regime, an optimal repetition rate was found at 500 kHz instead of 250 kHz. This result might be connected to the prevailing absorption at this wavelength of the mineral phase of the bone tissue—that is, the hydroxyapatite—which, as an inorganic matrix, is more thermo-resistant than collagen and hemoglobin. Moreover, the presence of water might lead to a conversion of the thermal load into an explosive ablation mechanism, leading to a higher ablation rate at this repetition rate with respect to results obtained at 250 kHz [38]. Finally, slightly different behavior is observed in the UV regime, where the ablation rate does not show any specific evolution with respect to the scanning speed, and it is accompanied by high calcination levels already when processing at 250 kHz and at the lowest scanning speed. In this regime, where the main absorption is driven by the collagen and hemoglobin content, the thermal load is simply too high to preserve the health of the bone tissue; regardless, the process parameters and ablation rates remain limited.

With respect to the laser-induced calcination of the bone tissue, results show that the shorter the wavelength, the easier the tissue carbonizes and calcines as a consequence of the laser interaction (Figure 5d–f). This observation is linked to the different absorption regimes of the different bone tissue components in the three wavelength regimes and their proneness to carbonization. For each set of process parameters, EDX analyses were carried out on ablated areas and non-ablated areas on the same piece of bone in order to evaluate the variation in the elemental composition with respect to its specific reference. The main components of the bone tissue (carbon C, oxygen O, magnesium Mg, calcium C and phosphate P) were evaluated in terms of atomic weight %. During the carbonization process, the combustion of the organic components of the bone tissue leads to a strong increase in the content of C. Once the amount of organic components of the bone becomes negligible at the end of the calcination process, only the mineral phase of the bone tissue remains (hydroxyapatite and mineral salts) [39], which can be identified by the typical grey-white color of the ablated areas and correlates with a strong decrease in the content of C [27]. Shades of other colors were observed, such as light pink, light yellow and light blue, which reveal different degrees of the laser-induced calcination of the bone tissue [28].

In the IR regime, laser-induced calcination of bone tissue takes place only at high cumulative energy per unit of surface—that is, at a high repetition rate and high spatial overlap between successive laser pulses. As previously mentioned, the absorption at this wavelength is driven by water and hydroxyapatite, which is less prone to calcination with respect to the organic components. The efficient absorption by the water likely leads to an explosive ablation mechanism, leading to reduced thermal accumulation effects [40].

In the visible regime, the highest ablation rate is obtainable. Nevertheless, ablation at this wavelength is accompanied by higher calcination of the ablated areas with respect to the IR regime. At 515 nm, around 39% of the analyzed ablated areas can be defined as calcined after the laser process, against around 11% for the IR regime. This observation is likely due to the strong absorption of hemoglobin, which is found throughout the whole volume of the cortical bone at this wavelength: being one of the main components of its organic matrix, it is readily impacted by the thermal loads and carbonization phenomena. An interesting behavior is visible for all processing parameters and at a repetition rate of 500 kHz, where the highest degree of calcination is observed without noticeable impact on the evolution of the ablation rate. This particular interaction regime may be connected to a specific temperature of the bone tissue reached at this repetition rate, which may induce a particular ablation regime.

**Figure 6:**
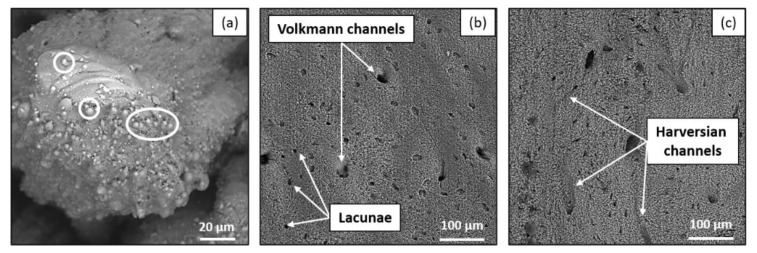
SEM images of laser-ablated bone tissue at (**a**) 515 nm, 1000 kHz, 4000 mm/s, 6.27 W, (**b**) 515 nm, 250 kHz, 4000 mm/s, 6.27 W and (**c**) 1030 nm, 250 kHz, 1000 mm/s, 6.27 W. White circles identify thermal effect-induced spherical microparticles while white arrows show Harversian channels, Volkmann channels and lacunae.

The laser-induced calcination of the bone tissue is readily visible by SEM analyses (Figure 2c, Figure 3c, Figure 4c and Figure 6a). The damage of the laser-irradiated tissue is visible as large micrometric cracks; spherical micro- and nano-particles appear at the top of these micrometric structures which are typical of laser-induced thermal mechanisms (Figure 6a). On the contrary, it is possible to observe that, in the case of ablation without calcination, the bone tissue still presents its characteristic elements, such as the Harversian and Volkmann canals (which are meant to contain and interconnect blood vessels), as well as lacunae (which are meant to contain osteocytes), as shown in Figure 6b,c. The presence of these kinds of biological systems after the ablation is fundamental for the fast recovery and the eventual regeneration of the tissue.

## 5. Conclusions

In this work, industrially available fs laser sources were employed to maximize the ablation rate of porcine femur to obtain undamaged laser ablated bone tissue within an upscaling approach of the process. A maximum ablation rate of 0.66 mm^3^/s was obtained when processing with a wavelength of 515 nm and repetition rate of 250 kHz. To the best of our knowledge, this is the highest value of ablation rate of porcine femur upon fs laser ablation currently reported in the literature. For the three investigated wavelength regimes, the upscaling of the process is possible up to a certain repetition rate and average power, over which thermal effects on the bone tissue are too important not to profoundly damage it. A high degree of calcination is observed when the organic matrix drives the absorption phenomena. In the IR regime, where the main absorption is due to the mineral matrix, low calcination is obtained together with low ablation rates. Several improvements could be implemented in the processing approach, such as the possibility to monitor the ablation depth in real time in order to reposition the laser focal point within its focal depth and optimize the laser energy deposition throughout the complete duration of the process. In this context, several well-known methods could be employed, the most common being optical coherence tomography (OCT) monitoring systems [41], inline coherent imaging (ICI) techniques [42] and laser-induced breakdown spectroscopy (LIBS) detectors [43]. These systems allow deeper insights into the state of the bone tissue during ablation and can also record real-time changes in bone composition, thus providing a real-time indication of bone carbonization. The integration of automated systems may lead to the full exploitation of the fs laser technology in the frame of high-resolution surgery in the clinical environment.

## Figures and Tables

**Table 1:** Literature summary of ablation rates from ultra-fast laser ablation of bone tissue.

Ref.	Bone Type	Wavelength (nm)	Pulse Duration	Repetition Rate (kHz)	Average Power (W)	Ablation Rate (mm^3^/s)
[9]	Bovine	800	210 fs	1	0.2	0.2 × 10^−3^
[11]	Bovine	1030	500 fs	3	1	20 × 10^−3^
[7]	Porcine femur	1030	900 fs	20	5	0.15
[20]	NA	1030	240 fs	60	NA	0.05
[21]	Sheep shank	1030	230 fs	300	20	0.99
[6]	Porcine ribs	1064	8 ps	500	9	0.09
[22]	Bovine femur	532	25 ps	100	20	0.19
[23]	Bovine femur	1064	12 ps	600	NA	0.15

**Table 2:** Laser processing parameters for tests in IR, visible and UV regimes.

Repetition Rate RR (kHz)	Average Power P (W)	Scanning Speed v (mm/s)
IR regime (1030 nm)		
250	2–2.92–5.28–6.27	1000–2500–4000
500		
1000		
Visible regime (515 nm)		
250	2–2.92–5.28–6.27	1000–2500–4000
500		
1000		
UV regime (343 nm)		
250	2–2.92–3.9	1000–2500–4000

**Table 3:** Highest ablation rate values obtained for non-calcined bone tissue in the three wavelength regimes and related laser processing parameters.

Regime	P (W)	RR (kHz)	v (mm/s)	OL (%)	Ablation Rate (mm^3^/s)	Ratio C_BEFORE_/C_AFTER_
IR—1030 nm	6.27	500	4000	60	0.30	1.74
Visible—515 nm	6.27	250	4000	36	0.66	2.02
UV—343 nm	3.9	250	2500	60	0.20	2.48

## Data Availability

Data available on request due to privacy restrictions. The data presented in this study are available on request from the corresponding author.
